# A 2′FY-RNA Motif Defines an Aptamer for Ebolavirus Secreted Protein

**DOI:** 10.1038/s41598-018-30590-8

**Published:** 2018-08-17

**Authors:** Shambhavi Shubham, Jan Hoinka, Soma Banerjee, Emma Swanson, Jacob A. Dillard, Nicholas J. Lennemann, Teresa M. Przytycka, Wendy Maury, Marit Nilsen-Hamilton

**Affiliations:** 1 Iowa State University, Ames, IA USA; 2 Aptalogic Inc., Ames, IA USA; 3 National Center for Biotechnology Information, NLM, NIH, Bethesda, MD USA; 4 Dept. Microbiology and Immunology, University of Iowa, Iowa City, IA USA; 5 Present Address: Integrated DNA Technologies, Coralville, IA USA

## Abstract

With properties such as stability to long-term storage and amenability to repetitive use, nucleic acid aptamers are compatible with many sensing/transducing platforms intended for use in remote locations. Sensors with these properties are important for quickly identifying ebolavirus outbreaks, which frequently start in locations that lack sophisticated equipment. Soluble glycoprotein (sGP), an excellent biomarker for ebolaviruses, is produced from the same gene as the ebolavirus glycoprotein GP1,2 that decorates the surface of the viral particle and is secreted in abundance into the blood stream even during the early stages of infection. Here, we report the selection and properties of a 2′fluoro pyrimidine (2′FY)-modified RNA aptamer, 39SGP1A, that specifically binds sGP. We demonstrate by computational and biochemical analysis that the recognition motif of 39SGP1A is a novel polypyrimidine-rich sequence. Replacement of -F by -OH in the 2′ position of the ribose resulted in complete loss of affinity for sGP. The protein motif to which the aptamer binds requires an intact sGP dimer and binds to an epitope conserved between Ebola virus (EBOV) and Sudan virus (SUDV) sGP, the most divergent *Ebolavirus* species. This identifies 39SGP1A as an excellent option for integration on a sensor platform to detect ebolavirus infections.

## Introduction

The species *Zaire ebolavirus*, in the family of *Filoviridae*, is a highly virulent enveloped negative strand RNA virus that causes hemorrhagic fever with 40–90% fatality. Early detection is the key to controlling outbreaks and epidemics such as those that have occurred in the recent past. The GP gene of all *Ebolavirus* species (Reston, Taï Forest, Sudan, Zaire and Bundibugyo) encodes three glycoproteins. The surface exposed trimeric glycoprotein (GP1,2) is the minor product, generated as a result of occasional transcriptional slippage. The major translated product is soluble glycoprotein (sGP), which is conserved amongst the *Ebolavirus* species^1,2^. sGP shares its first 295 amino acids with GP1,2, but has a unique species-specific 28 amino acid carboxy terminus, and is secreted as disulphide-linked, glycosylated homodimers. sGP and GP1,2 share the same 3D structure in the region of their sequence identity^3^. Although, the role of sGP in pathogenesis is not yet established, some antibodies elicited during ebolavirus infection cross react with sGP and GP1,2. This observation suggests a role for sGP as a decoy for antibodies against GP1,2 on the virus particle^4^. Consistent with this hypothesis is the observation that sGP is secreted in abundance in the early stages of infection and its production is selected for *in vivo*^2,4^. Although an excellent choice as an ebolavirus biomarker, sGP has so far been overlooked for this purpose.

Aptamers are short nucleic acids (RNA or DNA) that are selected by an *in vitro* iterative selection protocol called Systematic Evolution of Ligands through Exponential Enrichment (SELEX)^5–7^. They can be chemically synthesized at relatively low cost and have been incorporated into a number of diagnostic sensor platforms^8^. For increased stability to nucleases, RNA aptamers are often chemically modified at the 2′ position with fluorine, amino, or methyl groups^9,10^. Here we describe the application of SELEX combined with Next Generation Sequencing (NGS) and analysis by AptaCluster^11^ and AptaGUI^12^ to select a 2′FY-RNA aptamer (39SGP1A) with high affinity and specificity for sGP. 39SGP1A binds EBOV and SUDV sGP tightly (Kd ~30 nM and 250 nM respectively) and does not bind human serum albumin or other proteins present at relatively high concentrations in serum. The requirement for structure in the binding of 39SGP1A to sGP is evidenced by the lack of affinity for sGP of the RNA (2′ OH) version of this 2′FY-RNA aptamer. The 2′F modification promotes the C3′ endo conformation in the ribose sugar, stabilizes the 2′FY-RNA structure and increases molecular hydrophobicity^13^.

In summary, we describe the selection and characterization of an aptamer with a novel 2′FY-RNA binding domain consisting of a poly2′F-U loop with GAGC in the stem. This aptamer (39SGP1A) binds tightly to sGP from two divergent *Ebolavirus* species and has potential as a bio sensing element for ebolavirus detection.

## Materials and Methods

### Nucleic acids

Oligonucleotides used in the study were synthesized by Integrated DNA Technologies (IDT; Coralville, IA) or the Iowa State University DNA facility. A DNA single stranded oligonucleotide library (487D) was synthesized by IDT with the following sequence: GCCTGTTGTGAGC‌CTCCTGTCGAA (53 N) TTGAGCGTTTATTCTTGTCTCCC. N symbolizes that an equimolar mixture of A, C, G and T was used during synthesis of the library. The following primers were used for PCR amplification: 484 (TAATACGACTCACTATAGGGAGACAAGAATAAACGCTCAA) and 485 (GCCTGTTGTGAGCCTCCTGTCGAA) to create dsDNAs that included a 5′T7 polymerase promoter for reverse transcription reactions. Other purchased DNAs were 5196 (AGAAAAATGCAATAAAAAA) and the DNA templates used for preparing 2′FY and 2′OH RNAs for this study (listed in section “Preparation of 2′FY-RNAs”). 39SGP1A 2′FY RNA (GGGCGCUCAAUUUUUUAUUGCAUUUUUCUUUGAGCGCCC) was purchased from IDT.

### Conversion of ssDNA SELEX library to dsDNA

To generate the starting 2′FY-RNA pool, an extension reaction was performed using primer 484 and the starting ssDNA pool. 10 reactions, each of 100 μL and containing 2 μM 487D pool, 3.3 μM 484 primer, 0.5 mM dNTP mix, 0.03 U/μL DNA Taq Polymerase (GenScript) in reaction buffer (50 mM KCl, 10 mM Tris-HCl (pH 8.55), 1.5 mM MgCl_2_, 0.1% TritonX-100) was incubated at 94 °C for 5 min, 65 °C for 15 min then 72 °C for 99 min using the Multi GENE II PCR minicycler. The generated dsDNA was resolved through a 2% agarose gel, purified using a Qiagen PCR purification kit, and quantified using a Nanodrop spectrophotometer ND1000 Spectrophotometer (Nanodrop Technologies).

### Protein reagents

SUDV sGP (Lot#1610002, Cat# 0570-001) was purchased from IBT Bioservices (Rockville, MD). EBOV sGP protein was produced in 15-cm plates of HEK 293 T cells by PEI transfection with 30 µg of the expression vector. The next day, media was replaced with Opti-MEM low-serum media containing 1 mM HEPES and 1% non-essential amino acids. Supernatant was collected at 48 and 72 hours following transfection and filtered through a 0.45 µm filter and frozen at −80 °C until purified.

Supernatants were thawed and concentrated by centrifugation using Amicon Ultra-15 Centrifugal Filter Units with 10 kDa molecular weight cut off (MWCO). sGP-6xHis present in the concentrated supernatants was bound to and eluted from cobalt-bound Dynabeads using the Dynabead His-Tag Isolation & Pulldown Kit (#10103D) protocol. Eluted samples were passed through Zeba Spin Desalting Columns (#89882). The desalted material was washed 5–10 times with 400 µL of DPBS (Dulbecco’s phosphate buffered saline from Thermo Fisher) in an Amicon Ultra-0.5 mL Centrifugal Filter Unit. Final purified samples were diluted to desired concentration in DPBS. Purified sGP was analyzed by Coomassie Blue staining and western blotting using anti-GP monoclonal antibody, 21D10 (IBT Bioservices).

### 2′FY-RNA synthesis and purification during SELEX

2′FY-RNA was prepared by *in-vitro* transcription using a Durascribe^TM^ T7 transcription kit (Epicentre, Madison). About 2 nmoles of dsDNA from the pool generated by extension was incubated with 5 mM ATP, 5 mM GTP, 5 mM 2′F-UTP, 5 mM 2′F-CTP, 5 mM DTT, 5% DMSO, 0.2 U/μL T7 polymerase in a total volume of 2.8 mL and incubated at 37 °C for 4 h. The DNA mixture was then digested with 1 MBU DNAse I. The 2′FY-RNA was resolved through a 7 M urea 8% (19:1 acrylamide:bisacrylamide) gel in TBE buffer (89 mM Tris Borate, 2 mM EDTA, pH 9.1) to separate the residual NTPs and abortive transcripts. The transcribed RNA was eluted into TE buffer (10 mM Tris-HCl, 1 mM EDTA, pH 8.0) by incubating for 18 h at 37 °C then ethanol precipitated.

### Selection of 2′FY-RNA aptamers against sGP (soluble glycoprotein)

Selection of aptamers that bind sGP was carried out by SELEX (Systematic Evolution of Ligands by EXponential enrichment). The starting 2′FY-RNA pool was generated by *in-vitro* transcription using dsDNA (487D) pool as template. The 2′FY-RNA pool (1 × 10^15^ molecules; 2 nmoles) was incubated with 1 nmole sGP in Buffer A (137 mM NaCl, 2.7 mM KCl, 10 mM Na_2_HPO_4_, 2 mM KH_2_PO_4_, 5 mM MgCl_2_, pH 7.4) at room temperature (RT, which was 23–24 °C) for 1 h and the non-binding 2′FY-RNA sequences were removed by passing the mixture through a nitrocellulose membrane on which the protein and bound RNAs were captured. With each subsequent positive selection the ratio of 2′FY-RNA pool:sGP was increased to increase the stringency of selection. Increased stringency was achieved by maintaining a constant amount of 2 nmoles RNA (10 µM) in each round and decreasing the amount of protein to incrementally increase the ratio of RNA/protein from 2 to 3, 4, 6.25, 10 and 20, respectively in subsequent positive selection rounds. To remove nonspecific membrane binders, negative selections were performed by passing the pool through the nitrocellulose membrane in the absence of protein. Reverse transcription and polymerase chain reaction amplification (PCR) was used to generate DNA for subsequent selection rounds. Eight rounds of selection were performed, which included two negative and six positive selections. PCR (15 cycles) was performed with the reverse transcribed RNA product from the selected pool, 0.05 U/ul Taq polymerase, 1.5 mM MgCl_2_, 0.2 mM of each of the four deoxynucleotide triphosphates, 50 mM KCl, 10 mM Tris-Cl (pH 9.0), 0.01% Triton X-100 and 2 µM of each primer (oligonucleotides 484 and 485). The enriched pool from the 8^th^ SELEX round was cloned into the TOPO XL PCR cloning plasmid for sequencing, in addition PCR-amplified libraries were prepared for NGS using Illumina sequencing primers and appropriate bar coding.

### Bioinformatics Analysis and Data Preprocessing

Raw sequences were demultiplexed and assigned to their respective rounds using the preprocessing tool AptaPLEX^14^ with barcodes ATCACG, TAGCTT, GGCTAC, ATGTCA for rounds 1, 4, 6, and 8 respectively. Quality control was performed by allowing a maximum of one nucleotide mismatch between the reference and matched barcodes, and a maximum of two mismatches between the reference and matched primers (see SELEX library construction in the section “Selection of 2′FY-RNA aptamers against sGP (soluble glycoprotein)”). Any reads not conforming to these standards were excluded from downstream analysis. Aptamer families (clusters) were consequently extracted from each round using AptaCLUSTER. We performed a total of 10 iterations of locality sensitive hashing, sampling 40% of the indices in the randomized regions and allowing no more than 10 nucleotide mismatches between the seed sequence of a cluster to each remaining member. The resulting clusters and additional information were visualized and analyzed through the graphical user interface AptaGUI.

MEME was run with version 4.12.0 using default parameters whereas secondary structures were predicted using Mfold version 2.3 with standard parameters in the RNA mode.

### Preparation of 2′FY-RNAs for analysis

2′FY-RNA was prepared using a Durascribe transcription kit (Epicentre Technologies) with PCR-amplified dsDNA, which was prepared from DNA templates containing the same sequences as the resulting 2′FY RNAs. Templates used to make each of the 2′FY oligonucleotides used in this work are listed in parentheses after the number with the core T7 promoter sequence bolded and italicized. The resulting RNA sequences (with T and C in the DNA becoming 2′FU and 2′FC in 2′FY RNAs or U and C in 2′OH RNAs) are underlined. Single stranded DNAs 4789, 4797 and 5189 were amplified with primer 484 complementary to the 5′ end, resulting in RNAs with sequences complementary to the underlined sequences.

**4789** (GCCTGTTGTGAGCCTCCTGTCGAACAGGGTCGGTCAGCGCTAATTTTACTTGCTGAAGCTCACAGACTGCATTACGTTTGAGCGTTTATTCTTGTCTCCC;

**4797** (GCCTGTTGTGAGCCTCCTGTCGAATCGGCCTAGTTGTTTAATGTTAACAATAGATTGTTTAATCTCGTTGTGTTGCTTTGAGCGTTTATTCTTGTCTCCC);

**5011** (**TAATACGACTCACTATA**GGGCGCTCAATTTTTTATTGAGCGCCC)

**5012** (GGGCGCTCAATAAAAAATTGAGCGCCCTATAGTGAGTCGTATTA)

**RNA 5015** (GGGCGCUCAAUUUUUUAUUGAGCGCCC) was synthesized from a template that was created by annealing oligonucleotides 5011 and 5012.

**5179** (***TAATACGACTCACTATA***GGGAGACAAGAATAAACGCTCAATCTTTTTATTTTACTTTTTGTTGTAGAGCGCTTGTTTCTCTGAGTTGTAGCCATTCGACAGGAGGCTCACAACAGGC);

**5183** (***TAATACGACTCACTATA***GGGAGACAAGAATAAACGCTCAATT TTTTATTGCATTTTTCTTTGAGCGTCATTGATACCCTGTTTCCTTGAGCACTTCGACAGGAGGCTCACAACAGGC); **5189** (AGGAAACAGGGTATCAATGACGCTCAAAGAAAAATGCAATAAAAAATTGAGCGTTTATTCTTGTCTCCC);

**5192** (***TAATACGACTCACTATA***GGGAGACATAAACGCTCAATTTTTTATTGCATTTTTCTTTGAGCGTCATTGTGTTTCCTTGAGCACTTCGACAGGAGGCTCACAACAGGC);

**5193** (GCCTGTTGTGAGCCTCCTGTCGAATAGCTCAAGGAAACAGGGTATCATTCTTGTCTCCCTATAGTGAGTCGTATTA);

**5197** (***TAATACGACTCACTATA***GGGCGCTCAATTTTTTATTGCATTTTTCTTTGAGCGCCC); **5198** (GGGCGCTCAAAGAAAAATGCAATAAAAAATTGAGCGCCCTATAGTGAGTCGTATTA)

**RNA 5199** (GGGCGCTCAAUUUUUUATTGCAUUUUUCUUUGAGCGCCC) was synthesized from a template that was created by annealing oligonucleotides 5197 and 5198.

The transcribed 2′FY-RNA was resolved by electrophoresis through an 8% polyacrylamide–7 M urea gel, and eluted from the gel. The 2′FY-RNA was dephosphorylated by incubating for 1 h at 37 °C in 50 mM Tris-Cl pH 9.3, 1 mM MgCl_2_, 0.1 mM ZnCl_2_, 1 mM spermidine, 0.02 U/μL calf intestine phosphatase (Promega, Madison, WI) then extracted with phenol-chloroform, and precipitated with ethanol. The 2′FY-RNA was end-labeled by incubating with [γ^32^P] ATP and 1U/ul T4 polynucleotide kinase (New England Biolabs, Ipswich, MA), in 7 mM Tris-Cl,1 mM MgCl_2_, 0.5 mM DTT pH 7.6 at 25 °C. In preparation for the filter capture assay, the 2′FY-RNAs (2 nM) were denatured at 95 °C for 5 min. The 100 nt 2′FY RNAs such as 5183 were refolded in Buffer A at room temperature (RT) for 30 min. The 2′FY-RNA aptamer, (39SGP1A) was refolded by rapid cooling in ice water for 5 min then left on the bench for 10 min at RT.

The most stable MFold-predicted structures for all oligonucleotides investigated in this work are shown in Fig. S1.

### Filter capture assay

Refolded ^32^P-2′FY-RNAs were incubated at RT for 30 min with varying concentrations of sGP (10 nM to 1 μM) then the samples were passed through nitrocellulose membranes (HAWP 02500). The filters were immediately washed with 3 mL Buffer A, and the ^32^P quantified using a liquid scintillation counter (Tricarb 4910^TR^, Perkin Elmer). The data was fit to the formula: F = Fmin + (Fmax*L^n)/(L^n + Kd^n) to determine the Kd, where Fmax is the maximum bound fraction, L is the ligand concentration, Fmin is the starting minimum bound fraction, n is the Hill coefficient and F is the corrected fraction bound. The concentration of sGP is reported throughout as the monomer concentration and the Kd is calculated on this basis. Thus, the calculated Kd’s reported here should be halved when referring to the sGP dimer.

### UV crosslinking and electrophoretic mobility shift assay (EMSA)

Refolded ^32^P-2′FY-RNAs were incubated with sGP in Buffer A for 30 min at RT. The mixtures were analyzed for complex formation with sGP by resolution through a 6% native polyacrylamide gel run in THE buffer (33 mM Tris, 66 mM HEPES, 0.1 mM EDTA-Na, pH 7.5) at 200 V. The ^32^P was visualized using a phosphorimager.

RNA was *in vitro* transcribed in the presence of 2′F-UTP and 4-thio-uridine 5′triphosphate (Trilink Biotechnologies, San Diego, CA) at a molar ratio of 1:1 and 5′end-labeled with ^32^P as described previously. Three pmol of RNA was denatured at 95 °C for 5 min followed by immediately cooling on ice for 5 min. The RNA was folded in Buffer A for 10 min at RT. To link sGP to the 2′FY-RNA aptamer 5183, 3 pmol 2′FY-RNA was incubated with 15 pmol sGP in 15 μL Buffer A for 20 min at 23 °C for complex formation. This mixture was irradiated for 6 min and 12 min at 4 °C under a UV lamp (365 nm) with the samples placed 3 cm from the lamps. The UV-irradiated samples were then treated with proteinase K for 30 min and resolved through a 10% reducing SDS PAGE gel to separate the 2′FY-RNA-protein complex, free 2′FY-RNA and free protein. The ^32^P on the gel was visualized using a phosphorimager.

### Competition binding assays

Competition binding reactions were performed by incubating 3 μM sGP with 9 μM and 18 μM competitor 2′FY-RNA sequences for 30 min at RT in Buffer A. Following the pre-incubation period, ^32^P-labeled 39SGP1A 2′FY-RNA was added to the reaction mix to a final concentration of 150 nM and the samples were incubated for 15 min. The 2′FY-RNA-protein complexes were then UV crosslinked at 365 nm for 15 min at 4 °C. Crosslinked products were analyzed by resolving through a 10% SDS PAGE reducing gel, the gel was dried and imaged by Typhoon scanner. Quantification was performed using Image J software.

To establish if the poly2′F-U loop is important for sGP binding, ^32^P-39SGP1A 2′FY-RNA aptamer was incubated in the presence and absence of a 10-fold excess of unlabeled DNA oligonucleotide 5196 for 20 min. sGP was then added and the samples incubated for another 30 min followed by irradiation for 15 min (UV at 365 nm). The samples were resolved through a 10% reducing SDS PAGE gel to separate the 2′FY-RNA aptamer-protein complex from the free 2′FY-RNA and protein. The ^32^P on the gel was visualized using a phosphorimager.

### Statistics

When two values were subtracted one from the other or divided one by the other, the standard deviations were calculated to take into account both errors by calculating the square root of the sum of the squares of the two errors. Statistical comparisons were performed by a two-tailed t-test, or a one-way analysis of variance (ANOVA) with appropriate post-hoc analysis as indicated. A p value of < 0.05 was considered significant.

## Results

### Selection of 2′FY-RNAs with high affinities for sGP

**Figure 1:**
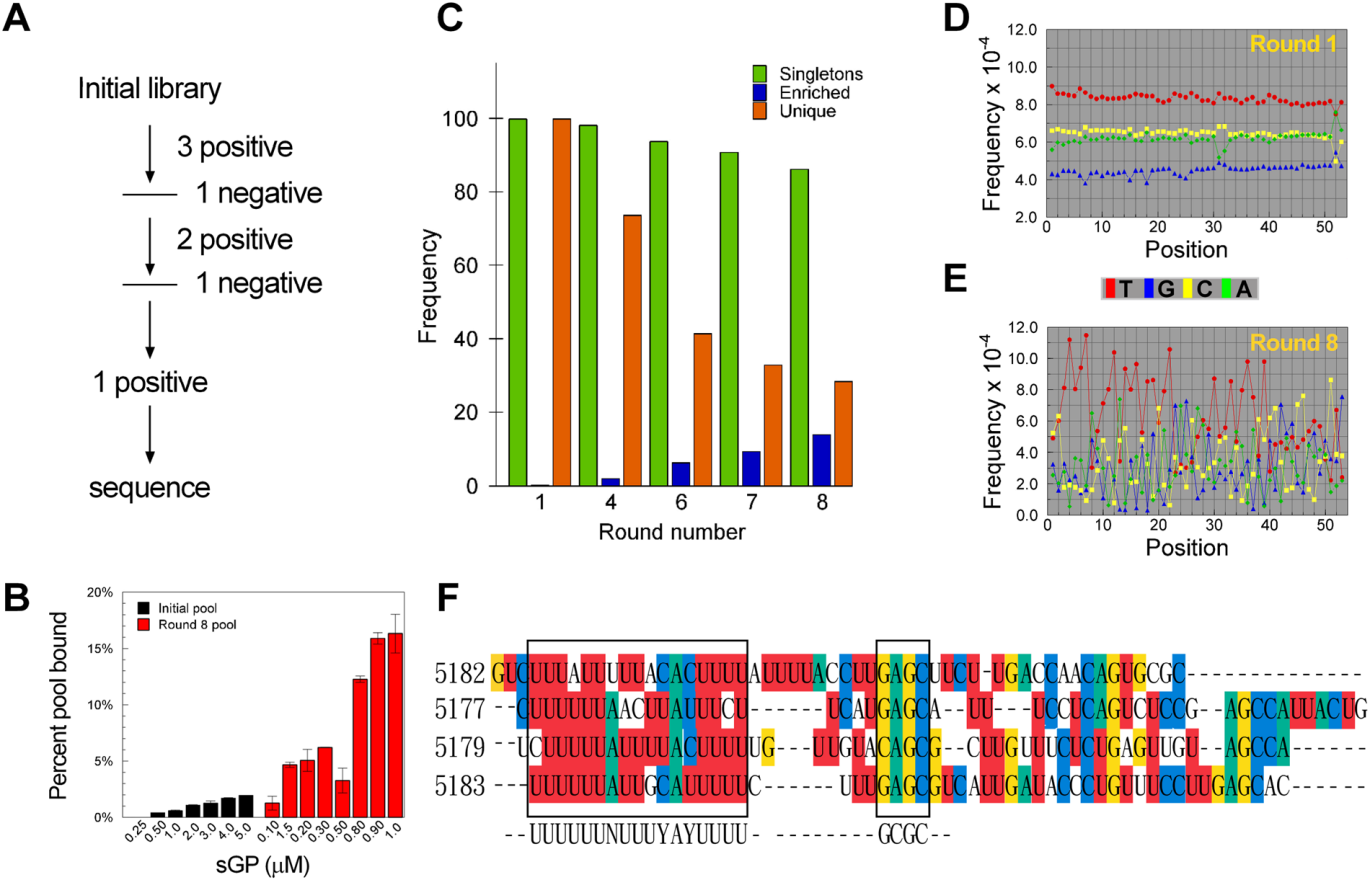
Next Gen Sequencing Analysis and Consensus Motif Identification (**A)** A flow diagram showing the SELEX protocol for selecting 2′FY-RNA aptamers with high affinity for EBOV sGP. (**B)** The percent of each round of selected 2′FY-RNA that bound EBOV sGP over the concentration ranges shown for the initial pool (60 nM 2′FY-RNA) and after the 6th positive selection (22 nM 2′FY-RNA). (**C)** The frequency of singletons, enriched and unique sequences for each SELEX round. (**D**,**E)** The frequency of base occurrence in each position for the 1st and 6th positive round pools showing evidence of sequence enrichment during selection. (**F)** A conserved sequence motif is identified among the top identified clusters.

2′FY-RNAs with high affinities for sGP were selected from an oligonucleotide pool, consisting of a central 53 nt random sequence region flanked by 25 bases 5′ and 3′ constant regions with a starting complexity of ~10^15^ molecules of 2′FY-RNA. The SELEX protocol followed a mathematically defined approach of starting with a relatively high molar ratio of 2:1 (2′FY-RNA: sGP) followed by harmonic reductions in the sGP concentration^15^ to increase the stringency of selections (Fig. 1A). To eliminate oligonucleotides with non-specific binding to the nitrocellulose membrane, rounds 4 and 7 were negative selections, which involved passage through the nitrocellulose filter, but no PCR amplification prior to the subsequent rounds 5 and 8 respectively. After 8 rounds of selection, the pools were analyzed by a nitrocellulose filter capture assay for evidence of high affinity binding populations. Whereas the initial pool showed a linear increase in the fraction bound with no signs of saturation up to 5 μM sGP, the binding curve of the oligonucleotide pool from the 8^th^ selection was biphasic with saturation achieved at ~150 nM and ~1 μM respectively suggesting selection of high and low affinity 2′FY-RNA binders (Fig. 1B). The filter binding results were corroborated by the results from electrophoretic mobility shift assay (EMSA) results using the pools from the 1^st^ and 8^th^ rounds with only the 8^th^ round pool demonstrating a mobility shift (Fig. S2). The pools of round 1, 4, 6 and 8 were sequenced resulting in the recovery of 254706, 265444, 194055, and 159246 potential aptamer sequences, respectively. The 8^th^ round pool was also sub-cloned and sequenced by TOPO-TA cloning, which resulted in 85 clones being analyzed.

### Sequencing analysis and identification of conserved sequence motifs

AptaCluster^11^ and AptaGUI^12^, an open source analysis tool for HT-SELEX data and its graphical user interface, were used for the NGS data analysis (Fig. 1C–E). The top 10 sequence clusters identified by AptaCluster from analyzing the NGS matched the top 10 sequence clusters obtained from TOPO TA cloning. These clusters are believed to represent families of aptamers that are closely related in sequence and derived during PCR due to the low stringency conditions used for transcription. The high-resolution NGS data showed a clear decrease in population diversity (the number of unique but possibly multi-copy sequences in a pool) and singletons (single-copy species) with increasing rounds of selection, whereas the aptamer families containing enriched sequences (species with increasing copy numbers through subsequent rounds) increased with each round (Fig. 1C). A comparison of the nucleotide frequencies at each position in the randomized region between the sequence population of rounds 1 and 8 also showed that selection had occurred (Fig. 1D,E). 10 oligonucleotides corresponding to the most frequent sequences in each of the 10 most abundant clusters were selected and analyzed using MEME^16^ for the presence of possible conserved primary structure motifs. Using this procedure, we identified a conserved sequence motif of a stretch of U’s and GAGC in the randomized region of the sequences (Fig. 1F). No other sequence structure motifs were found in these or in other enriched clusters examined.

### The poly2′F-U/GAGC sequence motif identifies a high affinity aptamer

**Figure 2:**
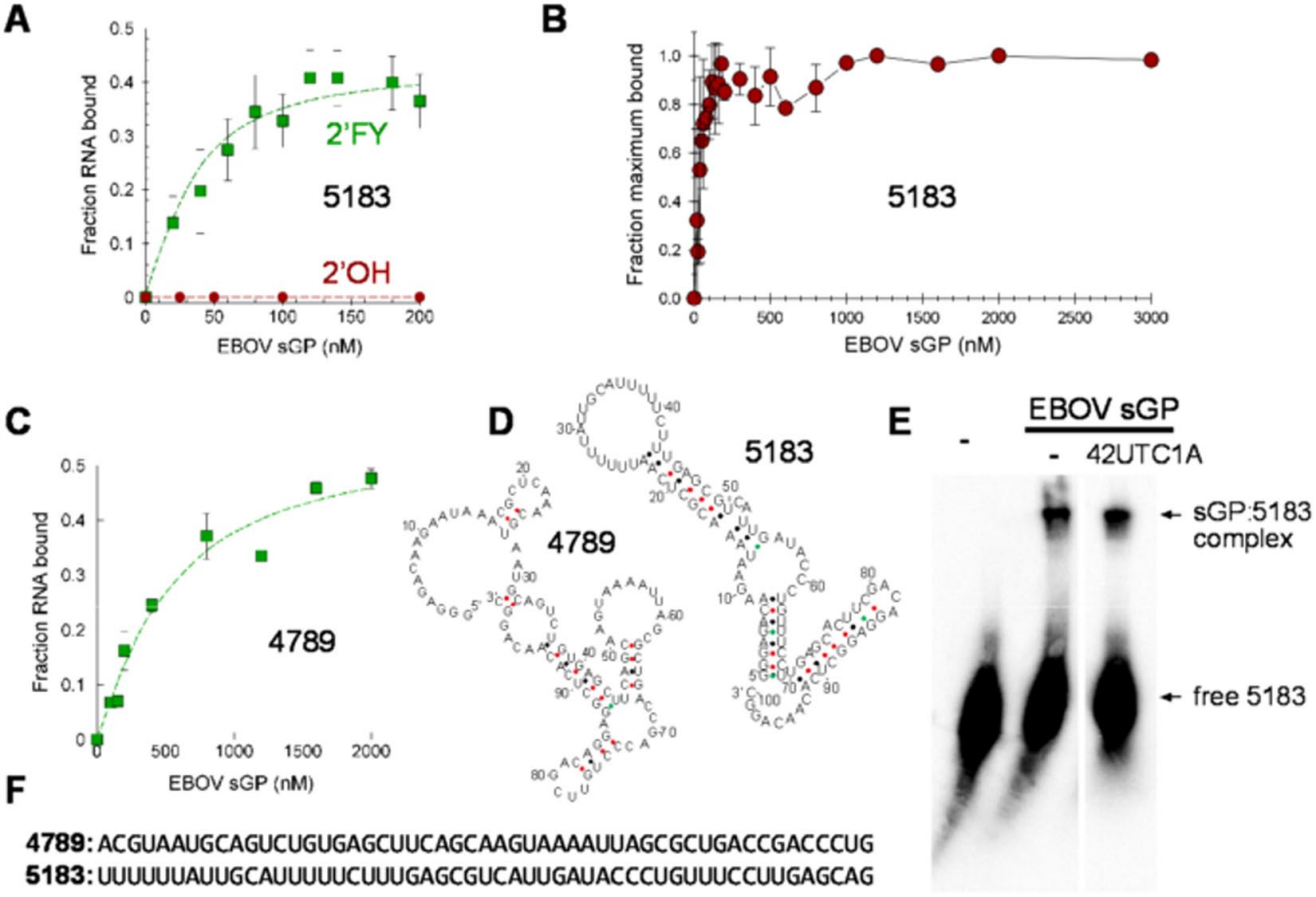
The Affinities of Selected 2′FY-oligonucleotides for EBOV sGP (**A)** The concentration dependence of EBOV sGP binding to 2 nM ^32^P-5183, which was in the form of a 2′FY-RNA (2′FY) or a RNA (2′OH). The estimated Kd was for the 2′FY-RNA was 54 nM. (**B**) An extended EBOV sGP titration up to 3 μM against 2 nM oligonucleotide^32^ P-5183 to validate saturation of the binding curve. This plot combines the results of 6 independent experiments, all having at least 3 concentrations higher than 150 nM. To normalize for the maximum fraction of RNA bound (as in panel 1 A), which is influenced by the efficiency of RNA folding, the data for each experiment was normalized to the maximum bound value prior to compiling and averaging the results from all experiments. (**C**) The concentration dependence of EBOV sGP binding to 20 nM ^32^P-4789. The estimated Kd was 530 nM. (**D)** The most stable Mfold-predicted secondary structures of oligonucleotides 4789 and 5183 (all predicted structures are in figures S4 and S5). (**E)** Electrophoretic mobility shift assay with 100 nM ^32^P-oligonucleotide 5183 in Buffer A. no addition; EBOV sGP, 2 μM EBOV sGP; 42UTC1A, 10 μM Lcn2 aptamer (42UTC1A). The two lanes are from the same gel but with irrelevant lanes removed (see Fig. S5 for the uncut image). (**F)** Sequences of the selected segments of RNAs 4789 and 5183. The sequences of these RNAs (excluding the primer sequences) are shown in (**D)**.

The affinities of 2′FY-RNA oligonucleotides 5183, 5177, 5179 and 5182 (Mfold structures in Fig. S3) that contain the poly2′F-U/GAGC sequence motif were compared with oligonucleotides 4789, 5181 from the top sequence cluster that did not contain this motif for their binding affinity to EBOV sGP. Saturation was achieved by 100 nM sGP in titrations against oligonucleotides containing the 2′F-U/GAGC motif, which is consistent with high affinity binding (Kds < 100 nM). The percent of the total RNA that bound sGP at saturation was used as an indication of the homogeneity of aptamer structure. By this criterion, 2′FY-RNA-5183, with the highest percent binding to sGP, was chosen and found to bind with a Kd of 54 nM (Fig. 2A). The amount bound reached saturation and did not increase further over a wide range of sGP concentrations (Fig. 2B), which is consistent with the interpretation that this oligonucleotide binds tightly to one epitope on sGP and does not bind with low affinity to another location on the protein. By contrast, oligonucleotides such as 4789, which did not contain the 2′F-U/GAGC motif, demonstrated much lower affinities (Kd = 0.5–1 μM) for sGP (Fig. 2C). The most stable structures predicted for 5183 and 4789 by MFold are shown in Fig. 2D,F. The same 5183 sequence in the form of RNA (with 2′OH) did not bind sGP (Fig. 2A). Replacement of 2′F-U with U but but retaining the 2′F-C in 5183 also resulted in loss of binding activity (Fig. S7). Thus, the 2′F-U modification is essential for 2′FY-RNA-5183 to bind sGP.

EBOV sGP binding by 2′FY-RNA 5183 was confirmed by electrophoretic mobility shift assay (EMSA, Fig. 2E). Binding to sGP was tested in the presence and absence of an unrelated RNA aptamer (42UTC1A) that recognizes mouse Lcn2^17^. 42UTC1A, in 10-fold molar excess, did not compete for 2′FY-RNA-5183 binding to sGP (Fig. 2E). These results are consistent with the hypothesis that the interaction of 2′FY-RNA-5183 with sGP involves a structural motif on the aptamer rather a basic patch on sGP capable of binding RNAs of many structures based largely on electrostatic interactions.

### Identification of the RNA epitope that binds sGP

**Figure 3:**
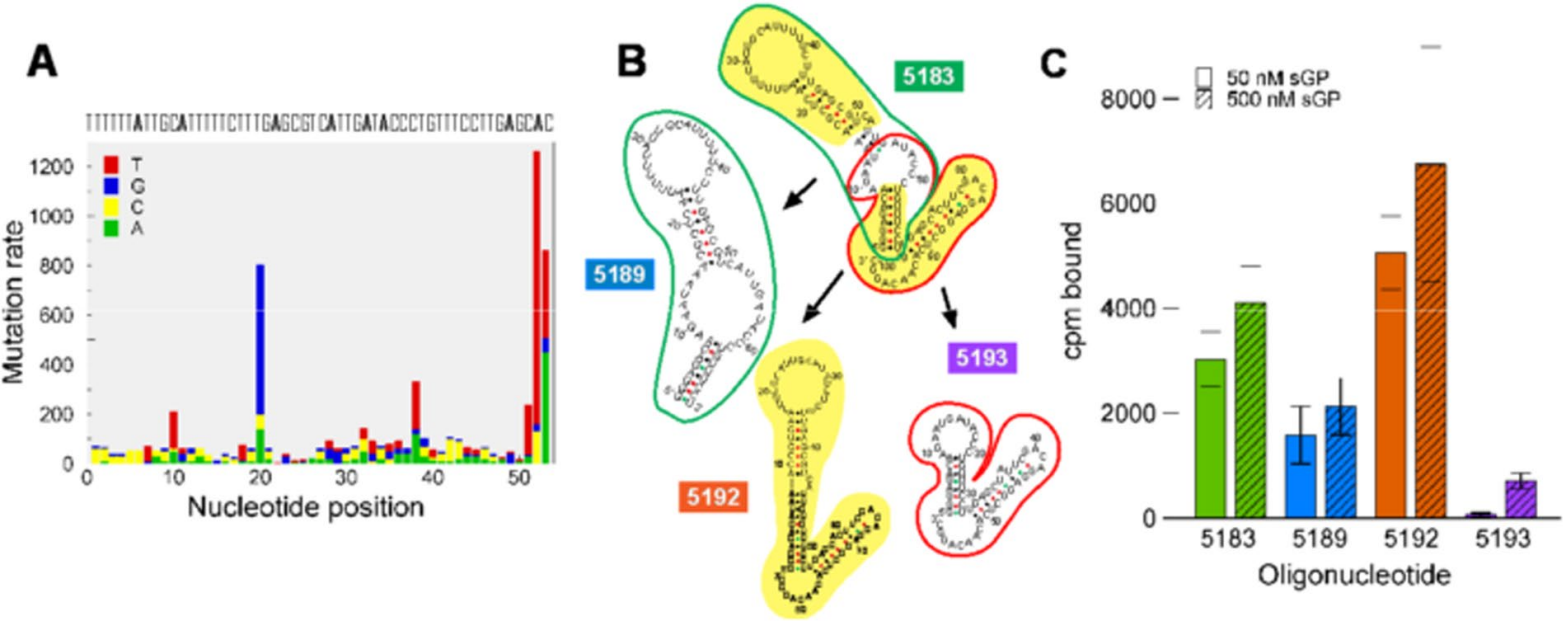
The aptamer binding domain (**A)** A mutation count chart created in AptaGUI to determine the mutation frequency for 5183 at each nucleotide position, (**B**) The Mfold-predicted secondary structures of truncations of 5183. The colored outlines reference the segments of 5183 that have been used to make 5189 (green) and 5193 (red) and the background highlighting shows the parts of 5183 that were used to construct 5192. **C)** Binding of 10 nM ^32^P-labeled oligonucleotides 5183, 5189, 5192 and 5193 to EBOV sGP at 50 nM (open bars) and 500 nM (hatched bars). The colors of the bars are matched with the colors behind the numbers in (**B)**.

Using AptaGUI to identify the RNA epitope and the minimal aptamer sequence for high affinity sGP binding by 2′FY-RNA-5183, we first compared oligonucleotide 5183 with several other oligonucleotides of the same aptamer family by calculating the base frequency of the cluster members at each nucleotide position in relation to 5183. This established the mutation frequencies (arising from errors during PCR) through the selections^12^. From this analysis, we identified the Mfold-predicted^18^ stem loop nearest to the 5′ end, which contains the poly2′F-U/GAGC sequence motif, as the most persistent sequence with the lowest mutation rate (Fig. 3A). Based on this analysis, truncations of 5183 (Fig. 3B) were prepared as 2′FY-RNAs and tested for binding to sGP (Fig. 3C). These results showed that the predicted 3′ stem loop is dispensable for sGP binding (5189) and by itself (5193) does not bind sGP. Removing the central bulge from 5183 resulted in a better sGP binding (5192), which suggests that the central bulge in 5183 is destabilizing.

**Figure 4:**
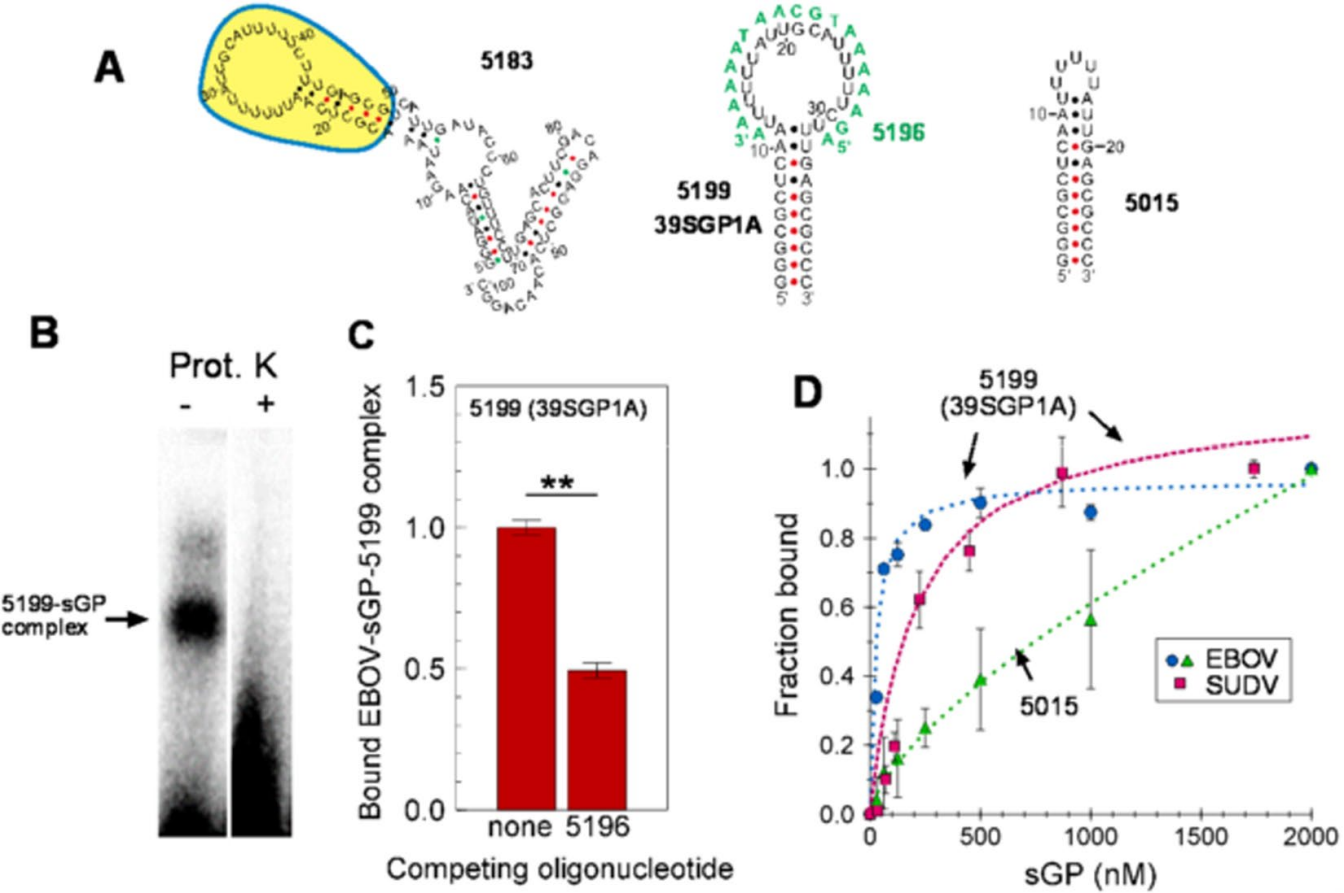
Identifying the Binding Epitope for EBOV sGP on the 2′FY RNA Aptamer (**A)** Mfold-predicted 2D structures of 5183 and the derived 5199 and 5015 oligonucleotides. The portion of 5183 that was taken as 5199 (39SGP1A) is highlighted in yellow. Oligonucleotide 5196, which is complementary to 5199, is shown in green lettering. (**B**) UV crosslinking of 200 nM ^32^P-39SGP1A to 2.5 μM EBOV sGP followed by treatment with or without proteinase K then analysis by EMSA. The two lanes are from the same gel but with irrelevant lanes removed (see Fig. S6 for an image of the uncut gel). (**C)** Binding of 0.5 μM ^32^P-39SGP1A to 10 µM EBOV sGP in the presence of 5 μM antisense oligonucleotide 5196, which is complimentary to the loop region of oligonucleotide 39SGP1A. **p < 0.05 (ANOVA). **(D)** Concentration dependence of EBOV and SUDV sGP binding to 10 nM ^32^P-39SGP1A and 10 nM ^32^P-5015. The estimated Kds from fitting were 27 nM for 39SGP1A with EBOV sGP and 240 nM with SUDV sGP, and 4 × 10^7^ for 5015 with EBOV sGP.

Of the oligonucleotides tested, the oligonucleotide with the minimal sequence from 5183 that bound sGP with highest affinity (2′FY-RNA oligo 5199) was taken as the sGP aptamer and named 39SGP1A (Fig. 4A). This aptamer was further tested for its ability to bind sGP by EMSA by which it was confirmed that the mobility-shifted ^32^P-2′FY-5199 RNA contained protein as it was lost after proteinase K treatment (Fig., 4B). Involvement of the poly2′F-U loop in sGP binding was tested by hybridizing 5199 with antisense oligonucleotide DNA-5196, which is complementary to the loop. The presence of a 10-fold molar excess of DNA-5196 over 39SGP1A resulted in 50% reduction in the sGP-2′FY-RNA shifted band in EMSA (Fig. 4C). An oligonucleotide containing a truncated sequence of 39SGP1A (2′FY-RNA-5015), in which the loop consisted of only 6 U’s, had no specificity for sGP (Fig. 4D). These results demonstrate that the polyU rich loop is required for specific sGP binding.

We also determined that 39SGP1A binds SUDV sGP, albeit with a 10-fold lower affinity, but still in the nanomolar range (Fig. 4D). Thus, 39SGP1A is a 2′FY-RNA aptamer that binds EBOV sGP with high affinity and recognizes an epitope on the protein that is related but is probably not identical to one within SUDV sGP, the most genetically distant of the *Ebolavirus* species.

### Specificity of 39SGP1A for sGP over other serum proteins

**Figure 5:**
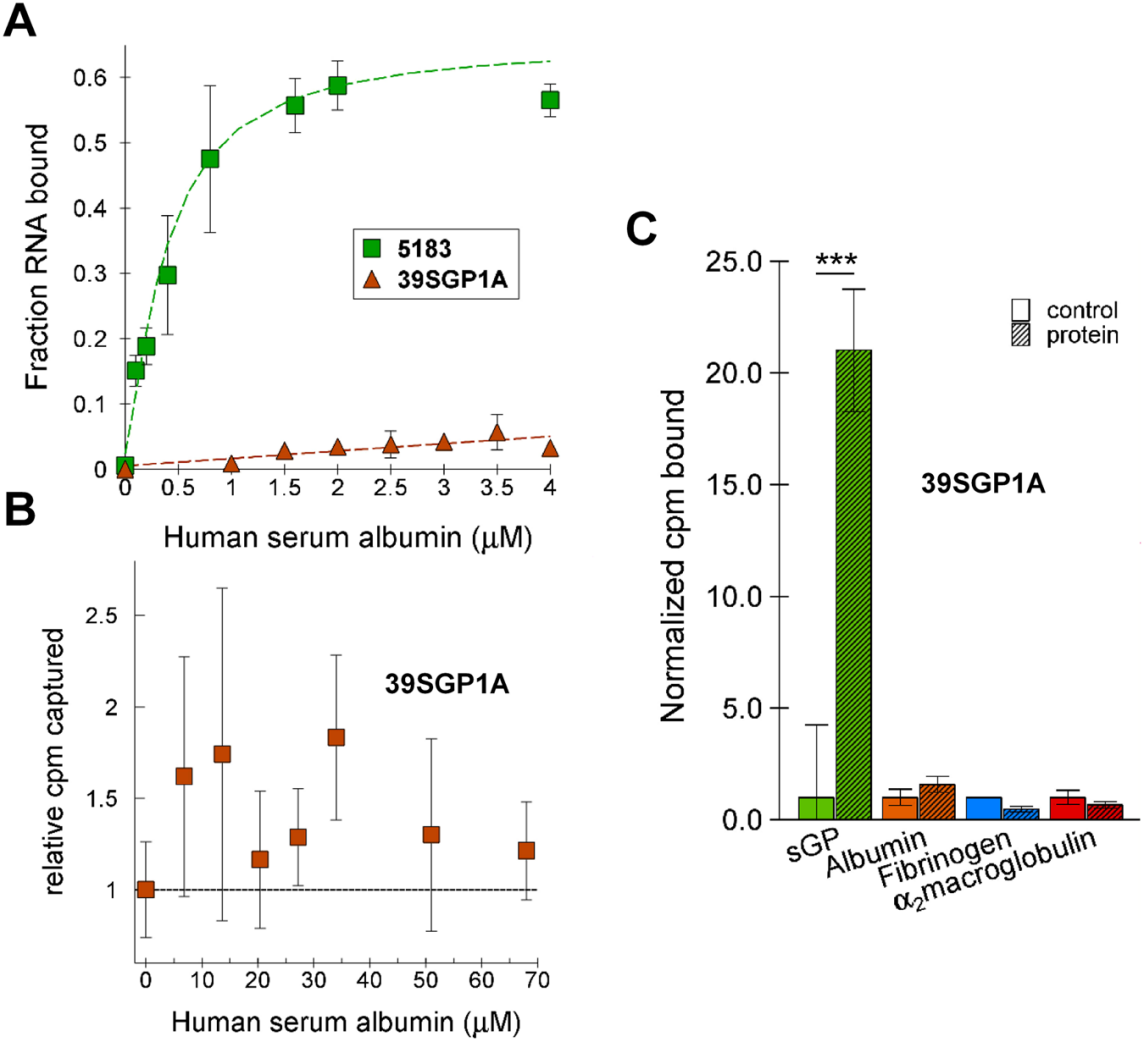
Specificity of Oligonucleotides 5183 and 39SGP1A for EBOV sGP (**A)** Binding of 2 nM ^32^P-2′FY-5183 aptamer and 10 nM ^32^P-39SGP1A to human serum albumin (HSA). (**B)** Binding of the 10 nM ^32^P-39SGP1A aptamer to HSA. All values are the average of duplicates with the error bars showing the estimated standard deviations (STDs). Where error bars are not seen, the STD is less than the size of the symbol. (**C)** Binding of 10 nM 39SGP1A to sGP (240 nM to 960 nM, N = 3, c.v. = 13%) human serum albumin (200 nM to 50 μM, N = 6, c.v. = 22%), fibrinogen (250 nM to 4 μM, N = 5, c.v. = 28%) or α_2_macroglobulin (205 nM to 5 μM, N = 5, c.v. = 21%). In each case, a range of protein concentrations was tested for which the N values are shown (an N of 1 is the result of a duplicate estimate at one protein concentration). The coefficients of variation (c.v.) for each average are consistent with the binding being at a plateau in these concentration ranges. The data used to create Fig. 5C is documented in Table S2. ***P < 0.0005 (two tailed Ttest).

To establish if 39SGP1A is suitable as a sensor for early diagnosis of sGP in patient serum, we tested its affinity for serum proteins. Human serum albumin (HSA) is highly abundant in blood, reaching concentrations up to 300 μM, whereas the concentration of sGP in serum is likely to be about 2 μM or lower^19^. Thus, the aptamer affinity for sGP must be more than 30-fold higher than for HSA to avoid HSA interference in detection of sGP by the aptamer. The original aptamer (oligo 5183) bound HSA with an estimated Kd of 400 nM, which is only about 10-fold higher than its Kd for sGP (Fig. 5A). However, the minimized aptamer, 39SGP1A, showed no binding to concentrations of HSA up to 68 μM (Fig. 5A–C). 39SGP1A also did not bind two other highly abundant serum proteins, α2 macroglobulin and fibrinogen (Fig. 5C).

### Characteristics of the sGP epitopes recognized by the selected 2′FY-RNA oligonucleotides

**Figure 6:**
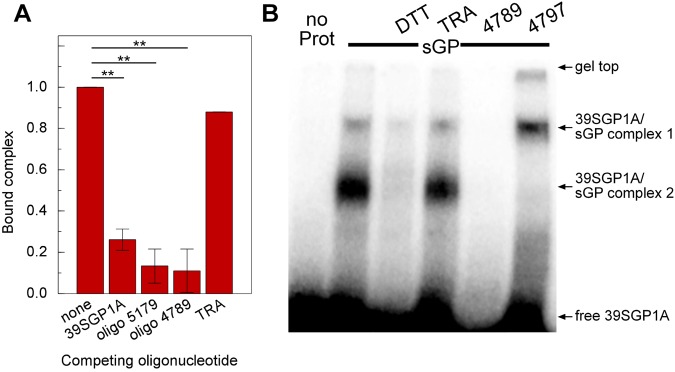
Analysis of the Protein Epitope for 39SGP1A (**A)** γ^32^P-39SGP1A (0.2 μM) was incubated for 15 min at 23 °C with a 100-fold excess of the identified oligonucleotides including the thrombin aptamer (TRA). The samples were stabilized by UV crosslinking and resolved by SDS-PAGE. The results from three experiments were quantified and compiled. **P < 0.05 (ANOVA). (**B)** An example of an EMSA from which some of the data in (**A**) was obtained. γ^32^P-39SGP1A was incubated with 3.8 μM sGP (-sGP-) and with no further additions (−), with 10 mM dithiothreitol (DTT) or with 20 μM thrombin aptamer (TRA), 4789 or 4797. After UV crosslinking, the samples were resolved by SDS-PAGE.

Several 2′FY-RNA oligonucleotide families were identified from the NGS results that are not homologous in sequence. Whereas members of the family identified by the poly 2′F-U/GAGC sequence motif bound sGP with high affinity, members of other families bound sGP with lower affinity, such as 4789 (Fig. 2C). We tested two of these oligonucleotides to establish if they bound to the same sGP epitope as 39SGP1A as demonstrated by their abilities to compete for binding of 39SGP1A to sGP (Fig. 6A). Oligonucleotide 5179 (in the same family as 5183), which contains the poly2′F-U-GAGC sequence motif (Fig. 1F), binds sGP with a similar high affinity to 5183. Oligonucleotide 4789 (in an aptamer family lacking the poly 2′F-U-GAGC sequence motif), bound sGP with lower affinity than 5183 (Fig. 2C). However, when present in 100-fold excess of 39SGP1A, 4789 competed as effectively with ^32^P-39SGP1A for binding to sGP as did 5179 (Fig. 6A). The control thrombin aptamer (TRA) did not effectively compete for binding to sGP. Mobility shift assays confirmed that 39SGP1A competed with oligo 4789 and not with the control thrombin aptamer for binding to sGP (Fig. 6B). These results suggest that sGP has a favored nucleic acid binding epitope, which binds aptamers of a variety of affinities, and that the binding affinity is determined by the tertiary structure of the 2′FY-RNA.

To test if binding to sGP by 39SGP1A required that the protein be a dimer, the binding reaction was performed in the presence and absence of dithiothreitol. These results showed that the protein epitope is lost when the dithiol-linked dimer was separated to form monomers (Fig. 6B) demonstrating that the minimum unit for 39SGP1A binding is the sGP dimer.

Another 2′FY oligonucleotide selected for sGP binding (4797) did not compete with 39SGP1A, but shifted the mobility of the sGP:39SGP1A complex to a higher position on the gel, suggesting that this oligonucleotide binds sGP in a different site from that bound by 39SGP1A.

## Discussion

In this study, we identified and characterized a 2′FY-RNA aptamer that binds the EBOV sGP dimer with high affinity and specificity and also binds SUDV sGP with 10-fold lower affinity. 39SGP1A is the first 2′FY-RNA or RNA aptamer reported to be specific for ebolavirus sGP. The choice of sGP as an aptamer target was motivated by the fact that it is secreted in abundance in blood early during infection, which makes it an excellent biomarker for ebolavirus infection^2^. sGP is a 82 KDa dimeric glycoprotein secreted by ebolavirus infected cells and is believed to be responsible for immune modulation during infection^4^. An aptamer that binds sGP could be incorporated onto a biosensor platform to detect early stages of ebolavirus infection.

The 2′F ribose modification was chosen for aptamer selection as it provides nuclease resistance and has been widely used in aptamer selections. After six rounds of positive selection and two rounds of negative selection from a starting pool of ~10^15^ 2′FY modified RNA oligonucleotides our results support the conclusion that the SELEX protocol resulted in the selection of two populations of oligonucleotides with high and low affinity for sGP, respectively. Some of the selected sequence clusters contained a conserved consensus sequence of poly U and GAGC, for which MFold-predicted secondary structures showed the poly U stretch of 18 nt in a loop region followed by GAGC’s in a stem. Oligonucleotides containing the polyU/GAGC motif bound sGP with high affinity (Kds = 20–54 nM). The 39 nucleotide sequence near the 5′ end of the oligonucleotide 5183 containing this motif was identified as the aptamer, 39SGP1A. Validation of this sequence as the aptamer included demonstrating the capture by UV-crosslinking of 39SGP1A and sGP in a single complex resolved by EMSA, its high affinity for the selection target, EBOV sGP, and the ability of a DNA oligonucleotide (5196), complementary to the polyU loop, to reduce 39SGP1A binding to sGP.

Aptamers from other clusters in the top 10 sequence populations that lacked the polyU/GAGC motif bound sGP with lower affinity. High and low affinity oligonucleotides competed for the same site in binding sGP but at least one oligonucleotide (4797) appears to bind to a second site on the protein. Thus, there are likely to be at least two epitopes on sGP that provide suitable targets for binding by 2′FY RNA aptamers.

The bias for T in the starting pool increased the probability that aptamers with high U content would be recovered. We investigated if sGP contained a region with high affinity for poly(U), which is present in some proteins^20–22^, by examining if 5183 RNA (prepared with 2′OH uracil and cytosine) bound sGP. The lack of affinity of 5183 RNA for sGP showed that the poly2′F-U is important for aptamer binding. This result and the observed differential affinity for EBOV and SUDV sGP is consistent with the hypothesis that the 2′F-U loop folds into a structure complementary to a sGP surface epitope.

The presence of 2′F-modified pyrimidines alters RNA structure by promoting the C3′ endo conformation of the ribose^23^, which is usually favored in RNA duplexes, increasing their helical stability. In a comparison of aptamers selected from libraries of RNA molecules containing 2′ amino- and 2′ fluoro-pyrimidines, the 2′FY-RNAs were found to have higher affinities than the 2′ NH_2_ modified aptamers^13^. The authors speculated that the increased affinity of 2′FY modified RNA is due to formation of thermodynamically stable helices and rigid structures in 2′FY-RNA compared with the 2′amino modified RNA^13^. However, X ray crystallography studies with 2′F modified and unmodified duplexes demonstrated that the presence of the 2′F modification does not alter the overall helical structure of the duplex^24^. The increased thermodynamic stability of the helices is due to the highly electronegative fluorine polarizing the nucleobases, which enhances Watson-Crick pairing. Osmotic stress and X ray crystallography studies showed that the presence of 2′fluorine reduced hydration of a duplex, increasing its hydrophobicity^24,25^. The additional helical stability and hydrophobicity of the 2′FY residues in 39SGP1A might promote a more stable structure, stronger intermolecular hydrogen bonding interactions and π-π stacking between the available 2′F uracils and hydrophobic amino acid side chains in sGP, thereby increasing aptamer affinity for the protein. In our study also, we observed higher retention to the hydrophobic nitrocellulose membranes of oligonucleotides containing poly2′F-U compared with those containing uridine.

39SGP1A binds dimeric, but not monomeric sGP and its binding site does not overlap with the epitopes recognized of any of three monoclonal antibodies antibodies (21D10, FVM-09, FVM-04; Fig. S8). The 39SGP1A binding site is also conserved between EBOV and SUDV, the most evolutionarily diverse ebolavirus species. These results suggest that the protein epitope for 39SGP1A is on a conserved surface on the protein, which is less likely to be lost on further evolution of the viral genome.

To be useful for detecting early ebolavirus infections, which would be performed with samples of patient blood^26^ or other body fluids, the 39SGP1A aptamer should not bind other serum proteins and should detect sGP in the presence of serum. Albumin, the most abundant serum protein is present in blood at concentrations as high as 300 μM. Although the selected oligonucleotide, 5183, from which 39SGP1A was derived, bound to HSA with a Kd of 400 nM, 39SGP1A had very low affinity for HSA. Thus, in this instance, truncating the oligonucleotide eliminated the options for 2′FY-RNA structures that could bind HSA. 39SGP1A also did not bind α_2_ macroglobulin and fibrinogen, two other serum proteins that could contribute to the background noise.

In summary, we report the selection of a 2′FY-RNA aptamer that binds EBOV and SUDV sGP with high affinity and specificity and demonstrate that the 2′F modification and poly2′FU loop is required for binding to sGP. This aptamer is an excellent candidate sensor for incorporating into a device for detecting evidence of an Ebola virus infection in biological matrices such as blood and serum.

## Electronic supplementary material

Supplementary information

## Data Availability

All data discussed in this report is available from the authors on request.
