# Supplementary material for: A 2′FY-RNA Motif Defines an Aptamer for Ebolavirus Secreted Protein
Source: Sci Rep. 2018 Aug 17;8:12373. doi: 10.1038/s41598-018-30590-8 (PMC6098113; doi:10.1038/s41598-018-30590-8)
Supplement: Supplementary file 1 — Supplementary information [file 41598_2018_30590_MOESM1_ESM.pdf]

Supplementary Material for “A 2’FY-RNA Motif Defines an Aptamer for Ebolavirus Secreted Protein”

Shambhavi Shubham, Jan Hoinka, Soma Banerjee, Emma Swanson, Jacob A. Dillard, Nicholas J. Lennemann, Teresa M. Przytycka, Wendy Maury and Marit Nilsen-Hamilton

Table of Contents

Table of Contents 1
Figure S1. Predicted 2D Oligonucleotide Structures 2
Figure S2. EMSA analysis of initial and final selected pools 3
Figure S3. MFold-predicted 2D Structures for oligonucleotides with the poly2’F-U/GAGC sequence motif 4
Figure S4. All MFold-predicted 2D Structures for 5183 5
Figure S5. All MFold-predicted 2D Structures for 4789 6
Figure S6. Full gel images for EMSA results shown in Figures 2 and 4. 7
Figure S7. The Ability of 5183 Containing Either 2’F-Uracil or Uracil to Bind sGP. 8
Figure S8. Competition between antibodies and selected oligonucleotides. 9
Table S1. Sequences of DNA Templates and RNA Products Used in this Work 10
Table S2. Data used to create Fig. 5C. 11

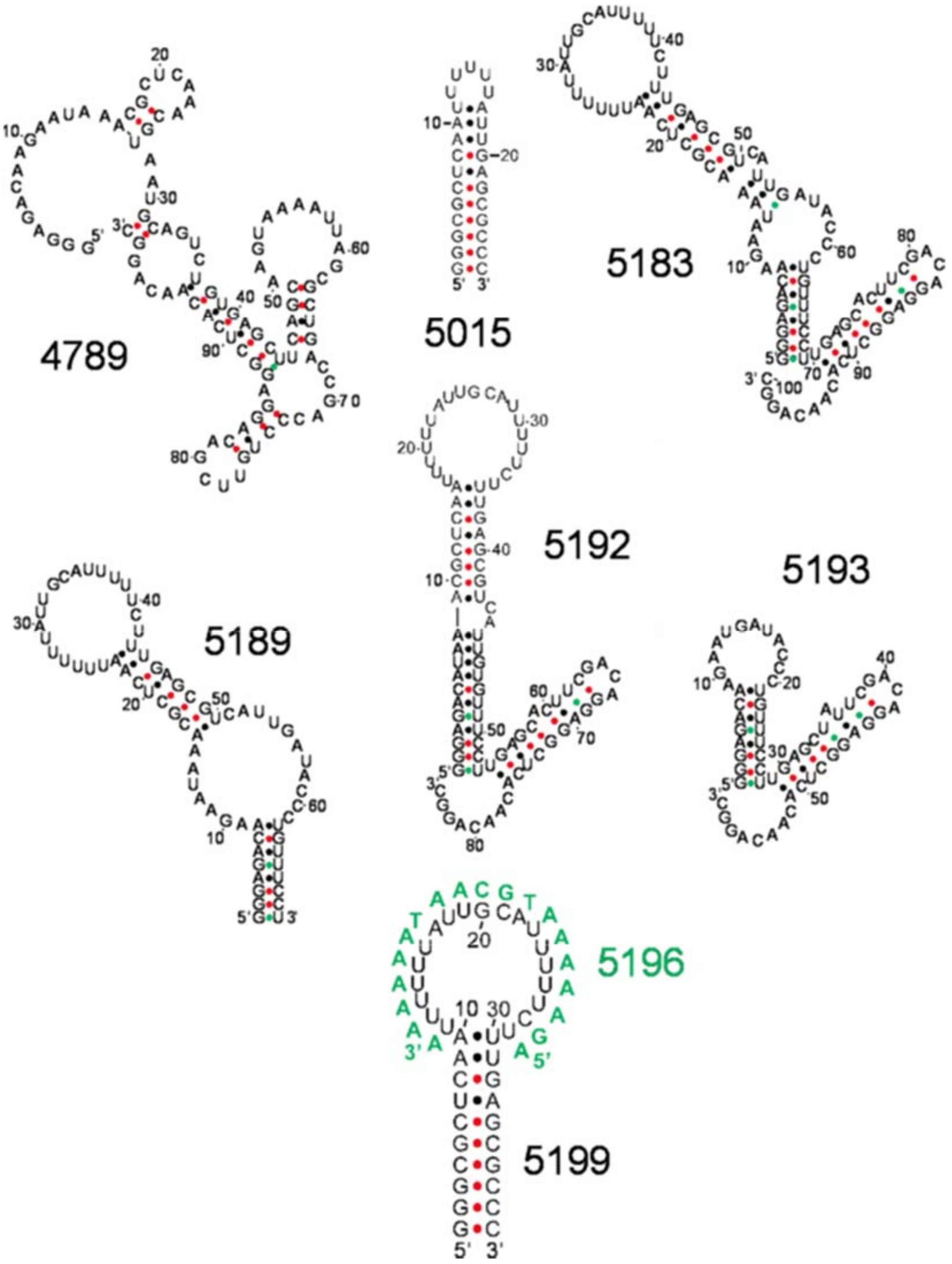

Figure S1. Predicted 2D Oligonucleotide Structures

Lowest energy structures proposed by MFold for oligonucleotides that were studied in this work.

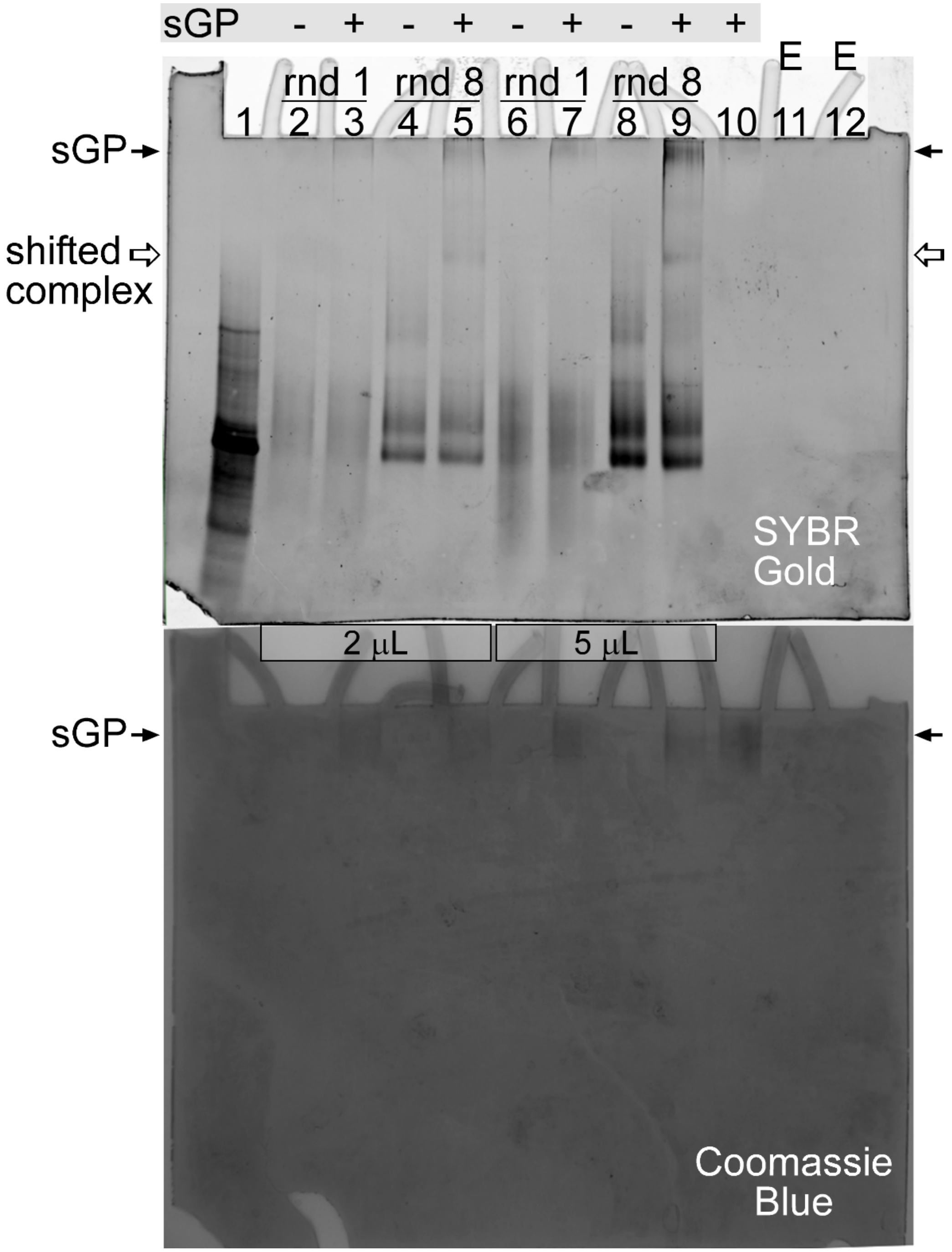

Figure S2. EMSA analysis of initial and final selected pools

The ability of the 1st and 8th round pools to be shifted by sGP was examined. 0.5 μM of each RNA pool was incubated with and without 5 μM sGP for 30 min at RT in Binding Buffer. Two and 5 μL of each mixture were loaded and resolved by electrophoresis. Position 11 and 12 were empty. Lanes 1 and 10 contained 4797 RNA or sGP, respectively. Round 1 (rnd 1) and round 8 (rnd 8) with or without sGP are in lanes 2-9. Two μL (lanes 2-6) and 5 μL (lanes 7-9) were loaded. The gel was first stained with SYBR Gold (top panel) and then with Coomassie blue (bottom panel). The positions of sGP and shifted RNA complex are identified.

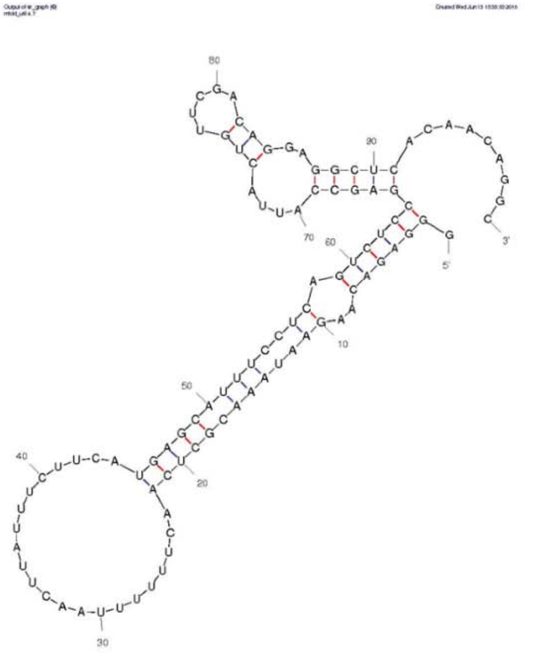

dG=-26.0 (initially -25.30) 5177

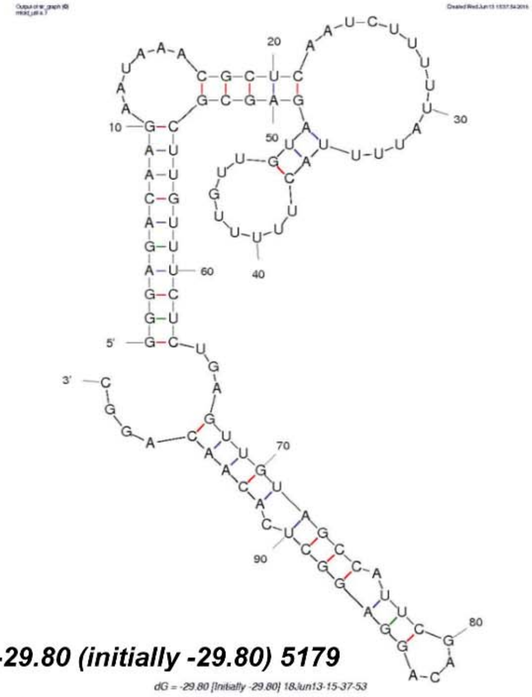

dG=-29.80 (initially -29.80) 5179

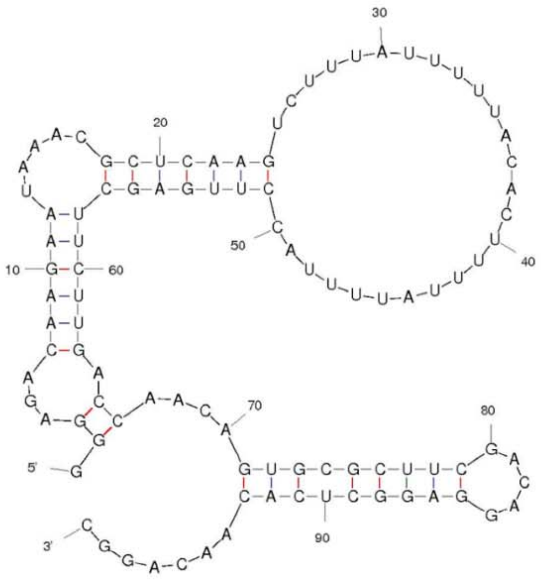

dG=-23.40 (initially -23.40) 5182

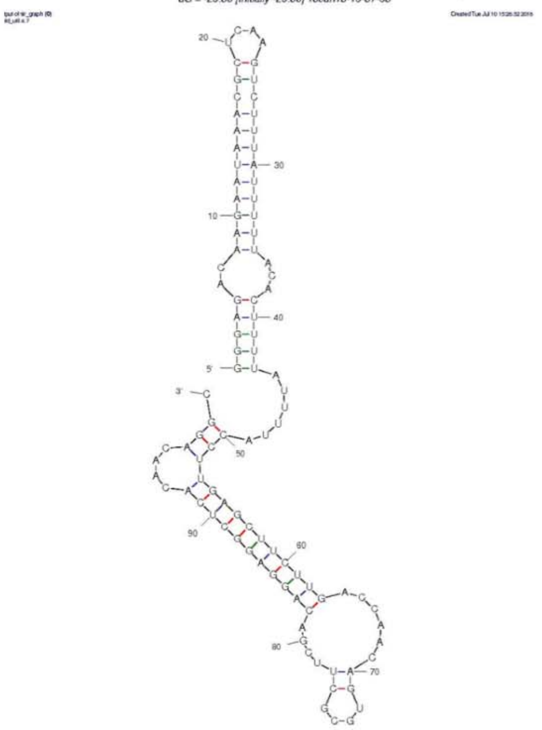

dG=-22.50 (initially -22.20) 5182

Figure S3. MFold-predicted 2D Structures for oligonucleotides with the poly2'F-U/GAGC sequence motif

Structures predicted by MFOLD using default parameters for oligonucleotides containing the poly2'F-U/GAGC sequence motif other than 5183. All predicted structures are shown.

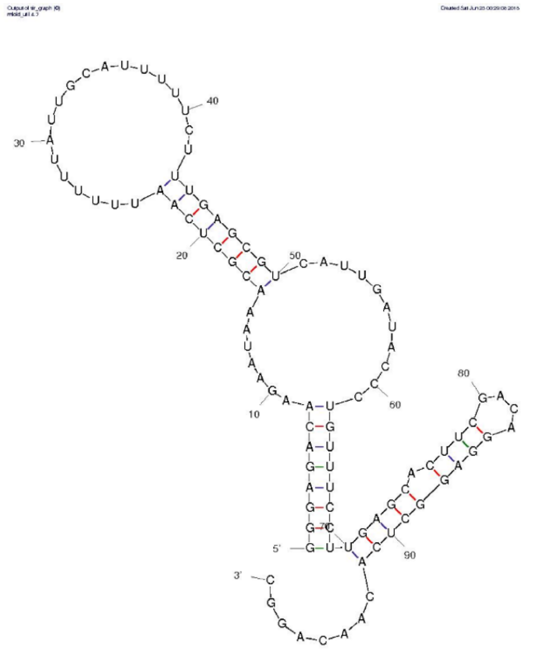

dG=-27.60 (initially -27.40) 5183

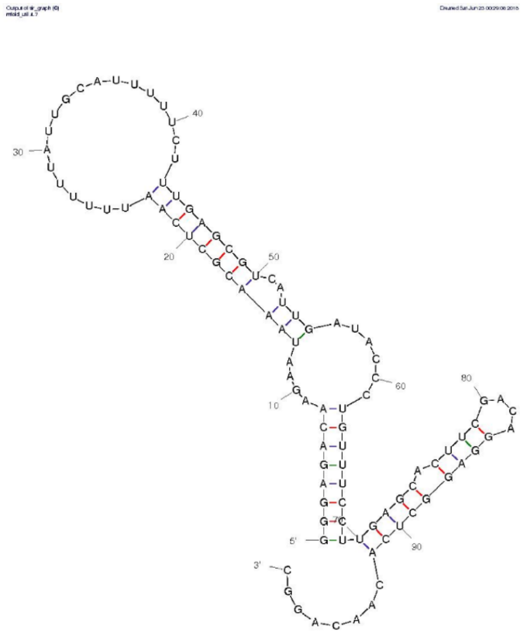

dG=-27.30 (initially -27.10) 5183

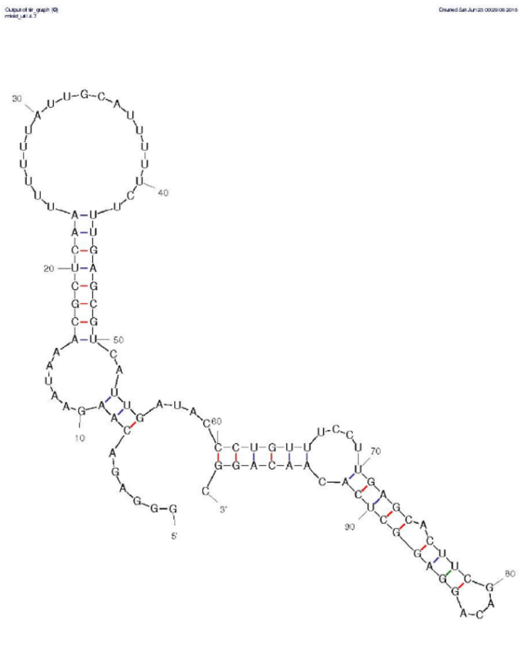

dG=-27.00 (initially -27.00) 5183

Figure S4. All MFold-predicted 2D Structures for 5183

The three structures predicted for 5183 by MFOLD using default parameters

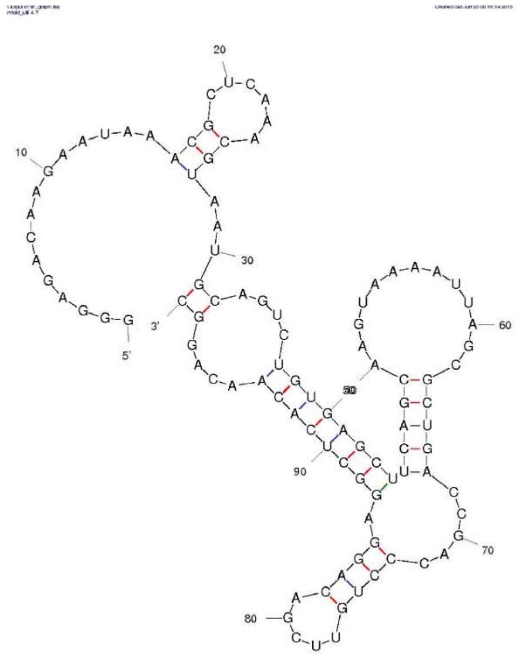

dG=-21.60 (initially -23.50) 4789

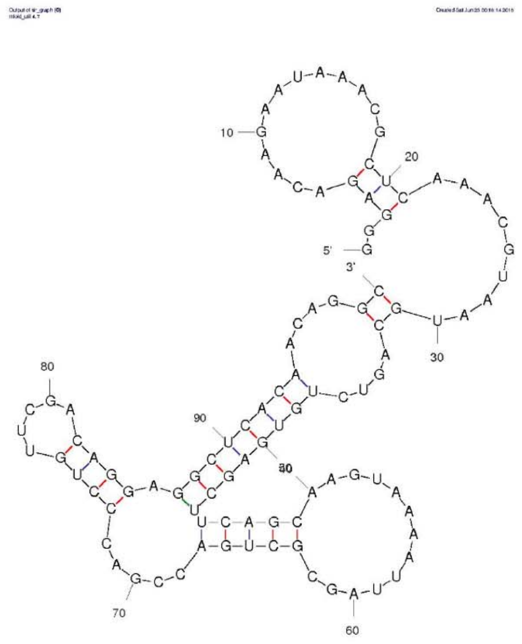

dG=-21.50 (initially -23.40) 4789

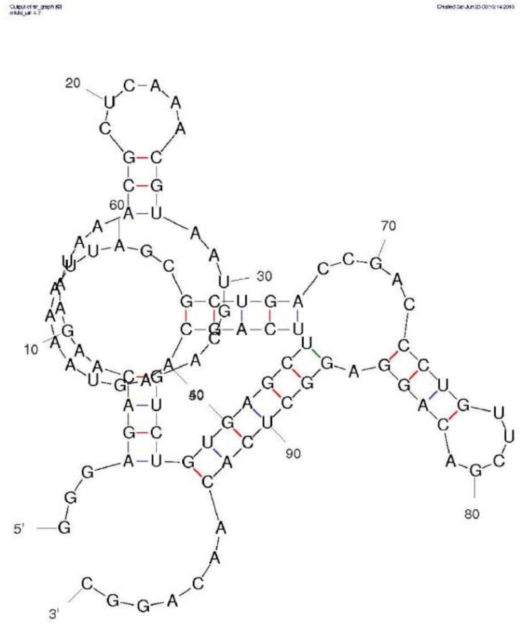

dG=-21.20 (initially -23.10) 4789

Figure S5. All MFold-predicted 2D Structures for 4789

The three structures predicted for 4789 by MFOLD using default parameters.

Figure 2

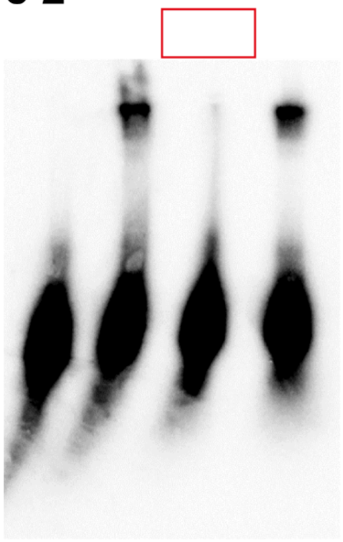

Figure 4

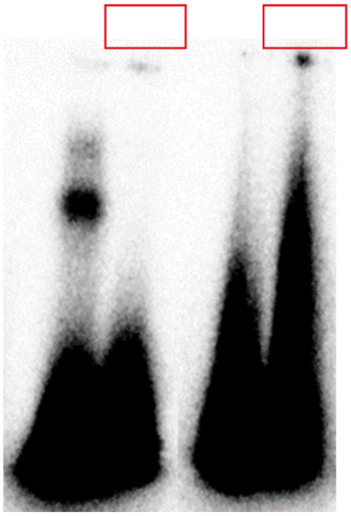

Figure S6. Full gel images for EMSA results shown in Figures 2 and 4.

Only the lanes relevant to the current work are shown in Figures 2E and 4B. Above, the uncut images are shown. The lanes deleted in creating figures 2E and 4B are identified in the figures by white vertical lines in place of the lanes that were removed, which are identified by the red rectangles immediately above the removed lanes.

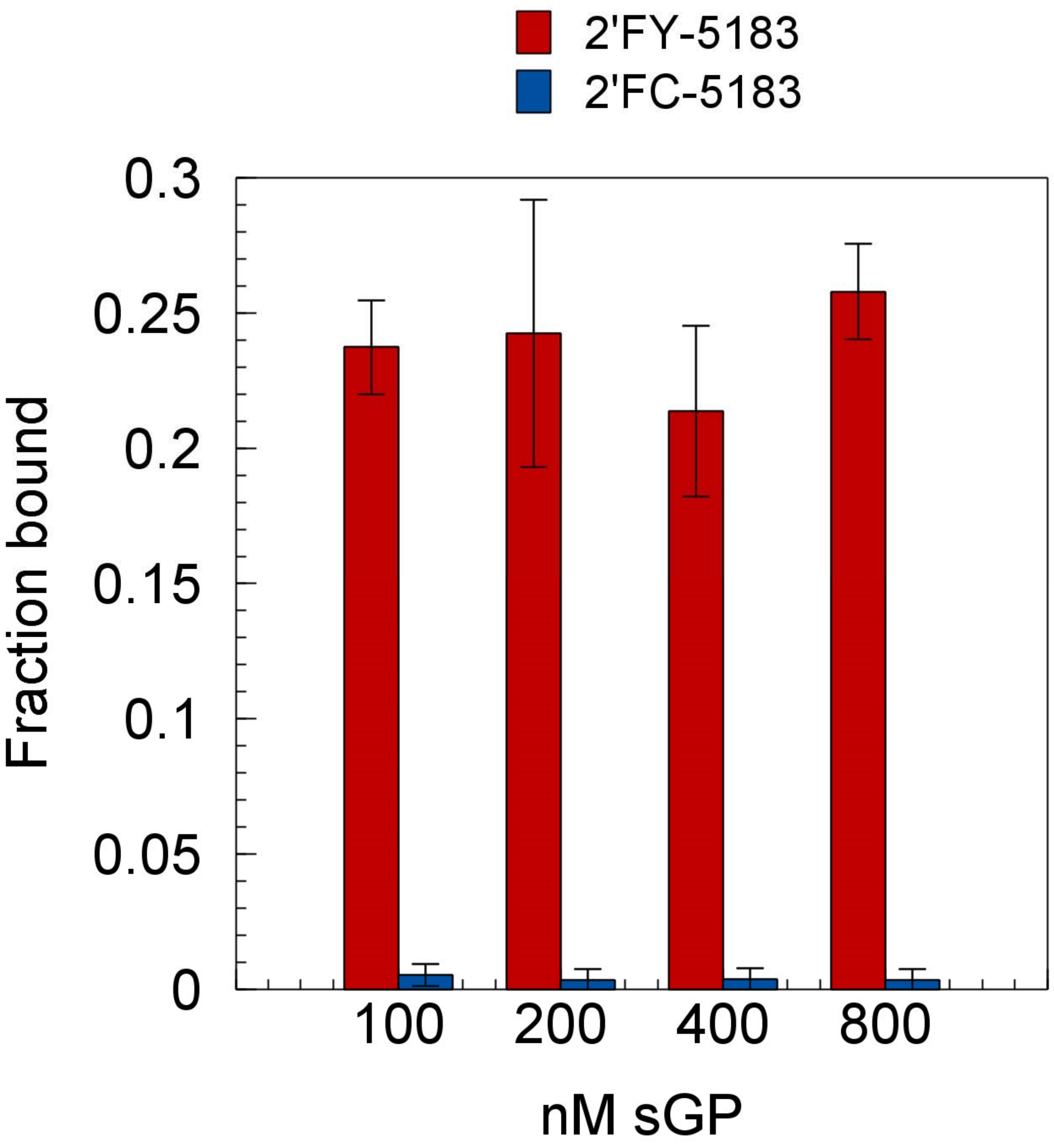

Figure S7. The Ability of 5183 Containing Either 2'F-Uracil or Uracil to Bind sGP.

Oligonucleotide 5183 was synthesized with both C and U with 2'F–ribose (2'FY-5183) or with only C attached to 2'F-ribose (2'FC-5183). These oligonucleotides (2 nM) were incubated with sGP (100, 200, 200, 800 nM) for 30 min at 23°C, then the protein-RNA complexes were collected by filtration and the filters counted. The fraction of total added ^{32}P-RNA is shown on the Y axis.

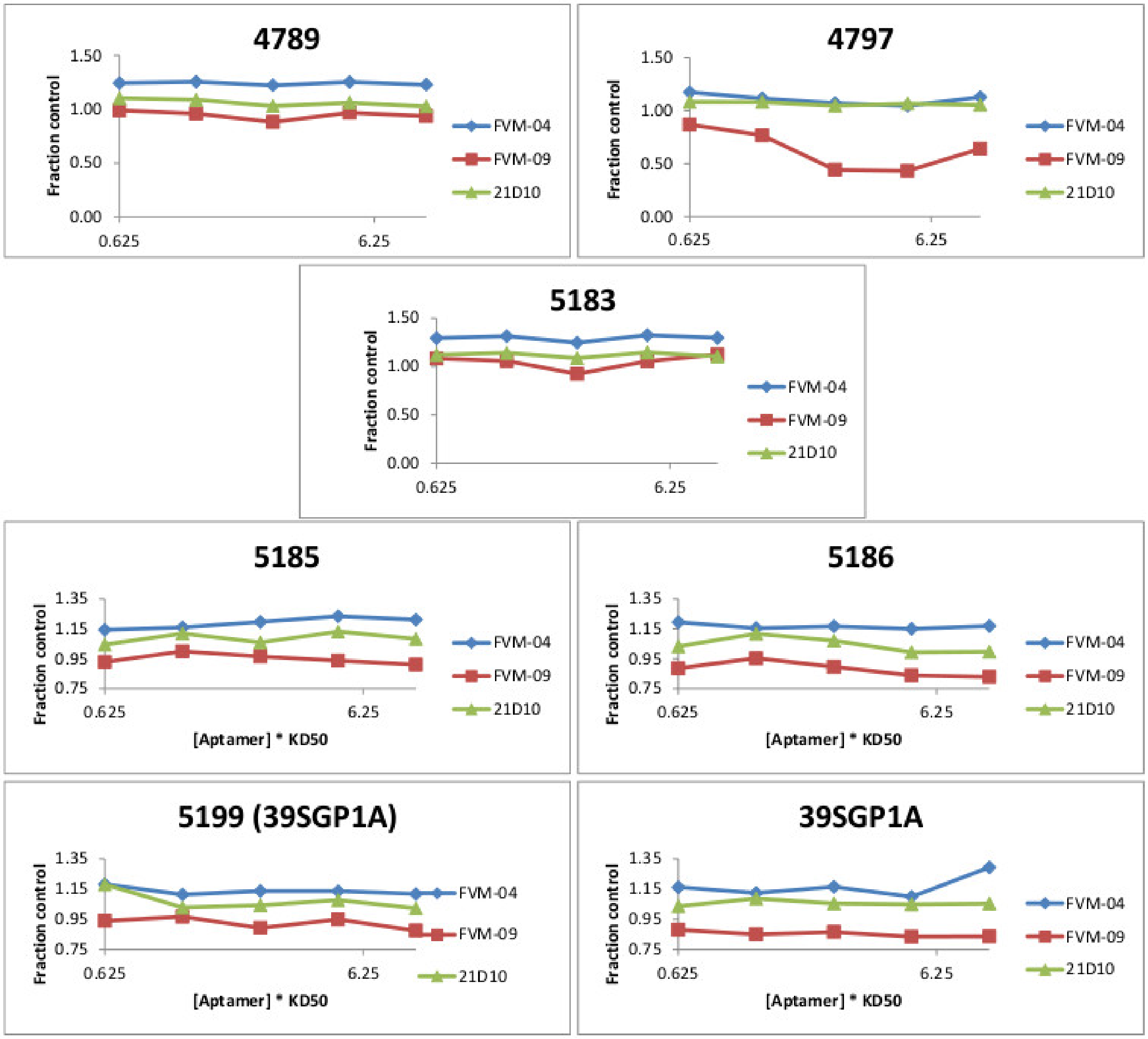

Figure S8. Competition between antibodies and selected oligonucleotides.

An ELISA assay was performed with sGP adsorbed to Immulon 2HB plates and each of the three antibodies 21D10(0.4 ug/mL), FVM-04 (0.06 ug/mL), and FVM-09 (0.007 ug/mL). For each antibody, the concentration of 2'FY-RNA aptamer was varied over a range of 0.625 to 10 times the estimated Kd for that 2'FY-RNA. The Kds used for this study were: 4789 (140 nM), 4797 (500 nM), 5183 (50 nM), 5185 (50 nM), 5186 (500 nM) 5187 (500 nM) 39sGP1A/5199 (50 nM). After incubation with secondary antibodies linked to HRP, the relative amounts of each antibody bound to sGP was determined using TMB Substrate Reagent Set (Sigma, Product No. T 8665) and measuring absorbance at 240 nm (A450). The data is reported as the A450 for each measurement (performed in duplicate) divided by the A450 for the control that lacked 2'FY-RNA.

#	sequence
4789 DNA Template	GCCTGTTGTGAGCCTCCTGTCGAACAGGGTCGGTCAGCGCTAATTTTACTTGCTGAAGCTCACAGACTGCATTACGTTTGAGCGTTTATTCTTGTCTCCC
4789 2'FY-RNA	GGGAGACAAGAAUAAACGCUCAAACGUAAUGCAGUCUGUGAGCUUCAGCAAGUAAAAUUAGCGCUGACCGACCCUGUUCGACAGGAGGCUCACAACAGGC
5183 DNA template	TAATACGACTCACTATAGGGAGACAAGAATAAACGCTCAATTTTTTATTGCATTTTTCTTTGAGCGTCATTGATACCCTGTTTCCTTGAGCACTTCGACAGGAGGCTCACAACAGGC
5183 2'FY-RNA	GGGAGACAAGAAUAAACGCUCAAUUUUUUAUUGCAUUUUUCUUUGAGCGUCAUUGAUACCCUGUUUCCUUGAGCACUUCGACAGGAGGCUCACAACAGGC
5189 DNA template	AGGAAACAGGGTATCAATGACGCTCAAAGAAAAATGCAATAAAAAATTGAGCGTTTATTCTTGTCTCCC
5189 2'FY-RNA	GGGAGACAAGAAUAAACGCUCAAUUUUUUAUUGCAUUUUUCUUUGAGCGUCAUUGAUACCCUGUUUCCU
5192 DNA template	TAATACGACTCACTATAGGGAGACATAAACGCTCAATTTTTTATTGCATTTTTCTTTGAGCGTCATTGTGTTTCCTTGAGC ACTTCGACAGGAGGCTCACAACAGGC
5192 2'FY-RNA	GGGAGACAUAAACGCUCAAUUUUUUAUUGCAUUUUUCUUUGAGCGUCAUUGUGUUUCCUUGAGCACUUCGACAGGAGGCUCACAACAGGC
5193 DNA template	GCCTGTTGTGAGCCTCCTGTCGAATAGCTCAAGGAAACAGGGTATCATTCTTGTCTCCCTATAGTGAGTCGTATTA
5193 2'FY-RNA	GGGAGACAAGAAUGAUACCCUGUUUCCUUGAGCUAUUCGACAGGAGGCUCACAACAGGC
5197 DNA template	TAATACGACTCACTATAGGGCGCTCAATTTTTTATTGCATTTTTCTTTGAGCGCCC
5198 DNA template	GGGCGCTCAAAGAAAAATGCAATAAAAAATTGAGCGCCCTATAGTGAGTCGTATTA
5199 2'FY-RNA	GGGCGCUCAAUUUUUUAUUGCAUUUUUCUUUGAGCGCCC
5011 DNA template	TAATACGACTCACTATAGGGCGCTCAATTTTTTATTGAGCGCCC
5012 DNA template	GGGCGCTCAATAAAAAATTGAGCGCCCTATAGTGAGTCGTATTA
5015 2'FY-RNA	GGGCGCUCAAUUUUUUAUUGAGCGCCC

Table S1. Sequences of DNA Templates and RNA Products Used in this Work

Sequences of the DNA templates and 2'FYRNA products are shown with the row containing the RNA sequence preceded by the sequence(s) for the DNA template(s). For the RNA, the 2'F-U is bolded in red and the 2'F-C is bolded in blue.

	values												values/zero control				
Protein	sGP			albumin			fibrinogen			alpha2 MG			sGP	HSA	FNG	a2MG	
(nM)	avg	std	c.v.	avg	std	c.v.	avg	std	c.v.	avg	std	c.v.					
0	9742	31536	324%	26739	9845	37%	68386	991	1%	127744			1.00	1.00	1.00	1.00	
240	175433	45675	26%										18.0				
250							27990	10608	38%						0.41		
325										105491	34367	33%				0.83	
480	227753	21226	9%										23.4				
500							30394	11945	39%						0.44		
650										89089	6526	7%				0.70	
960	210645	20240	10%										21.6				
1000							12924	8424	65%						0.19		
1300										86267	11458	13%				0.68	
2000							9372	9274	99%						0.14		
2600										85172	1222	1%				0.67	
5000				47909	24768	52%								1.79			
5200										55643	8591	15%				0.44	
10000				52062	34399	66%								1.95			
15000				32360	14194	44%								1.21			
20000				36555	10014	27%								1.37			
25000				55207	17098	31%								2.06			
37500				37003	19893	54%								1.38			
50000				34024	10112	30%								1.27			
													p=	3.95E-08	1.96E-05	2.18E-06	T test compared with sGP

Table S2. Data used to create Fig. 5C.

The data from four experiments using the filter capture assay are compiled. Images of radioactive 39SGP1A captured on filters were analyzed using ImageJ. Each data point is the average of duplicate values for the protein concentration shown. Standard deviations and coefficients of variation (c.v.) are shown. Areas in each well are shown for duplicate
